# A case report: Comorbidity of Rhinocerebral mucormycosis and pulmonary aspergillosis with challenging diagnosis

**DOI:** 10.3389/fmed.2024.1398714

**Published:** 2024-09-25

**Authors:** Qi Wang, YangYiYi Huang, HaiNa Ma, Guo-Kang Fan

**Affiliations:** Department of Otolaryngology, The Second Affiliated Hospital Zhejiang University School of Medicine, Hangzhou, China

**Keywords:** Rhinocerebral mucormycosis, *Rhizopus*, metagenomic next-generation sequencing, case report, amphotericin B, posaconazole

## Abstract

**Background:**

Mucormycosis is a rare opportunistic invasive fungal disease. Rhinocerebral mucormycosis (RCM) is clinically difficult to diagnose, and patients often die due to delayed diagnosis.

**Case description:**

A patient with concurrent pulmonary aspergillosis was diagnosed with RCM caused by *Rhizopus* through metagenomic Next-Generation Sequencing (mNGS). Despite comprehensive treatment including surgery, amphotericin B, and posaconazole, the patient tragically passed away. The treatment was delayed due to repeated cultures of secretions were negative and pathological examination could not clarify which fungus is infected.

**Conclusion:**

The clinical manifestations of RCM are not specific in the early stage, but the infection progresses rapidly. Therefore, early and accurate diagnosis is very important. mNGS is helpful for patients suspected of RCM, especially when conventional microbiology tests were negative.

## Introduction

Mucormycosis is a rare opportunistic invasive fungal disease, and *Rhizopus* is a common pathogen in mucormycosis (1). RCM refers specifically to a mucormycosis in which the fungus penetrates the nasal cavity through the skin-mucosa junction, spreads to the palate, sinuses, and orbit, and finally causes intracranial infection due to vascular invasion or bone destruction. Mucormycosis can occur in various parts, and the frequency of occurrence is in the order of RCM (46.8%), lungs, skin, and gastrointestinal tract (2). All-cause mortality rates for mucormycosis range from 22.9 to 80% (2–4). Lower mortality is seen with localized sinus (38.6%) or skin infection (22.9%), where earlier tissue-based diagnosis is often feasible and surgical debridement may result in cure (2). Culture of nasal secretions and pathological examination may fail to identify the specific fungal infection. On the contrary, mNGS has been increasingly utilized in the diagnosis of infectious diseases, particularly when conventional tests were negative or there is a high demand for timeliness in clinical practice.

Here we report one case of RCM due to *Rhizopus* infection, in which the diagnosis was eventually established with the help of mNGS. Microbiological culture of nasal secretion was negative and pathological examination cannot clarify which fungus is infected.

## Case presentation

**Figure 1:**
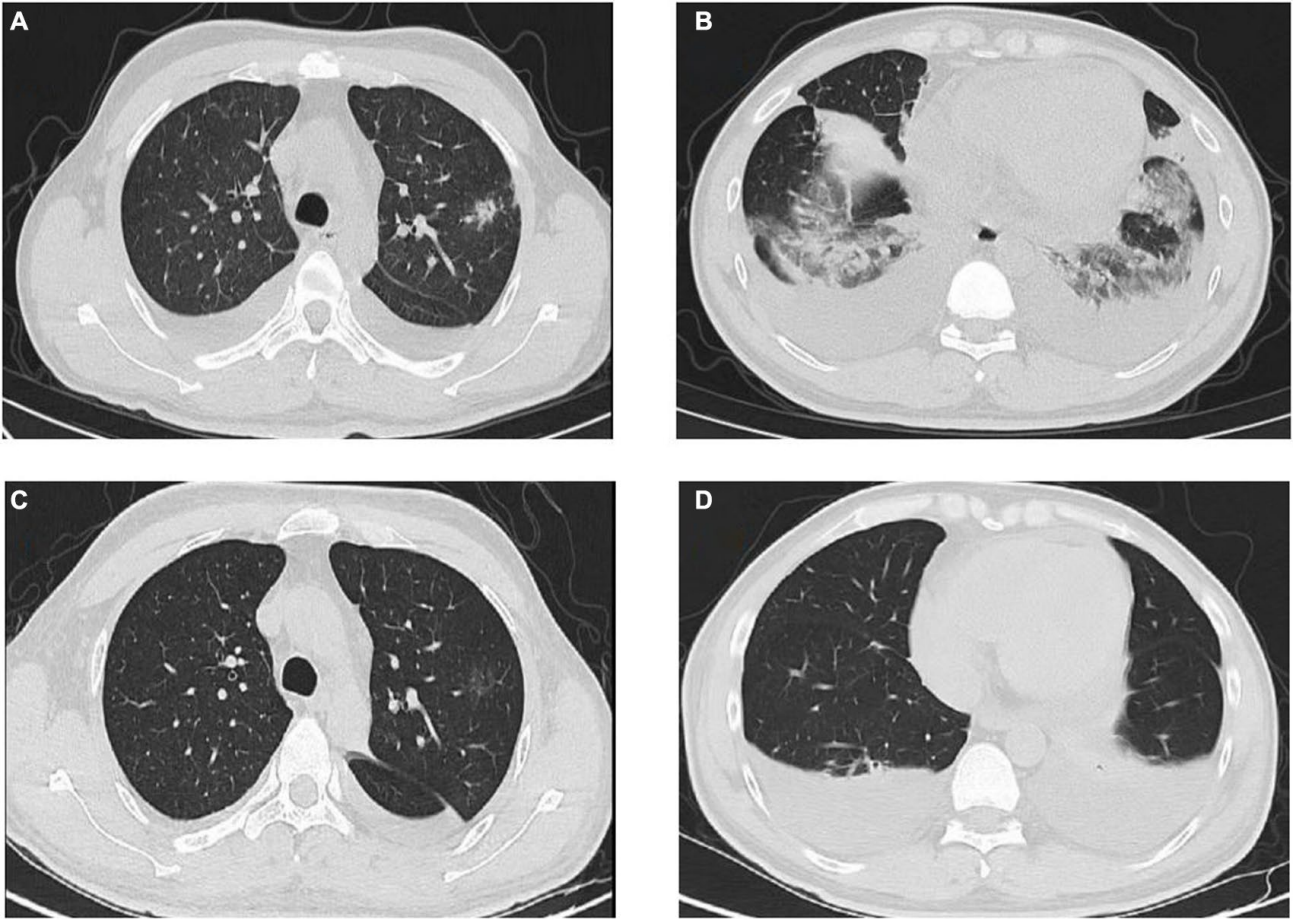
Comparison of before and after lung CT results. **(A,B)** February 22nd lung CT: Multiple solid nodules with exudation in the posterior segment of the left upper lobe of the lung, and exudation in the lower lobes of both lungs. **(C,D)** March 30th lung CT: The inflammatory nodules in the upper lobe of the lung were significantly absorbed and became lighter, and some were completely absorbed; bilateral pleural effusion.

**Figure 2:**
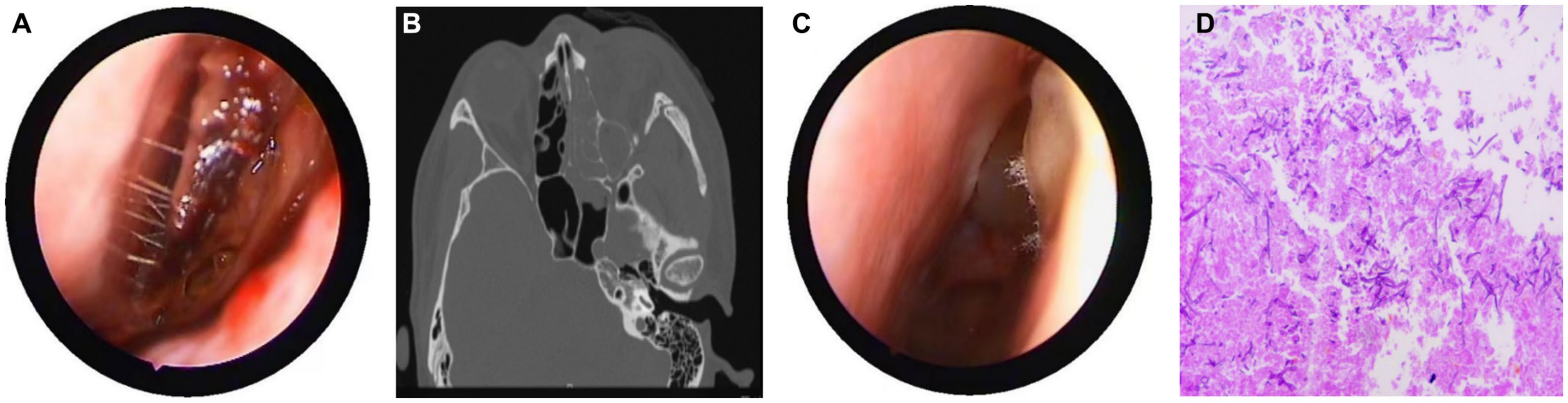
Nasal endoscopy, sinus CT, and pathological results. **(A)** Endoscopic examination showing pus in the left middle meatus and blood scab on the surface of the middle turbinate. **(B)** CT showed left ethmoid and frontal sinusitis, with thickening and swelling of the left medial rectus and inferior rectus muscles, and swelling of the soft tissues around the left orbit and cheek. **(C)** During the intraoperative nasal endoscopy, white hyphae were found in the left nasal cavity. **(D)** Postoperative pathology: a large number of fungal hyphae and spores were seen in the nasal and sinus mucosa.

**Figure 3:**
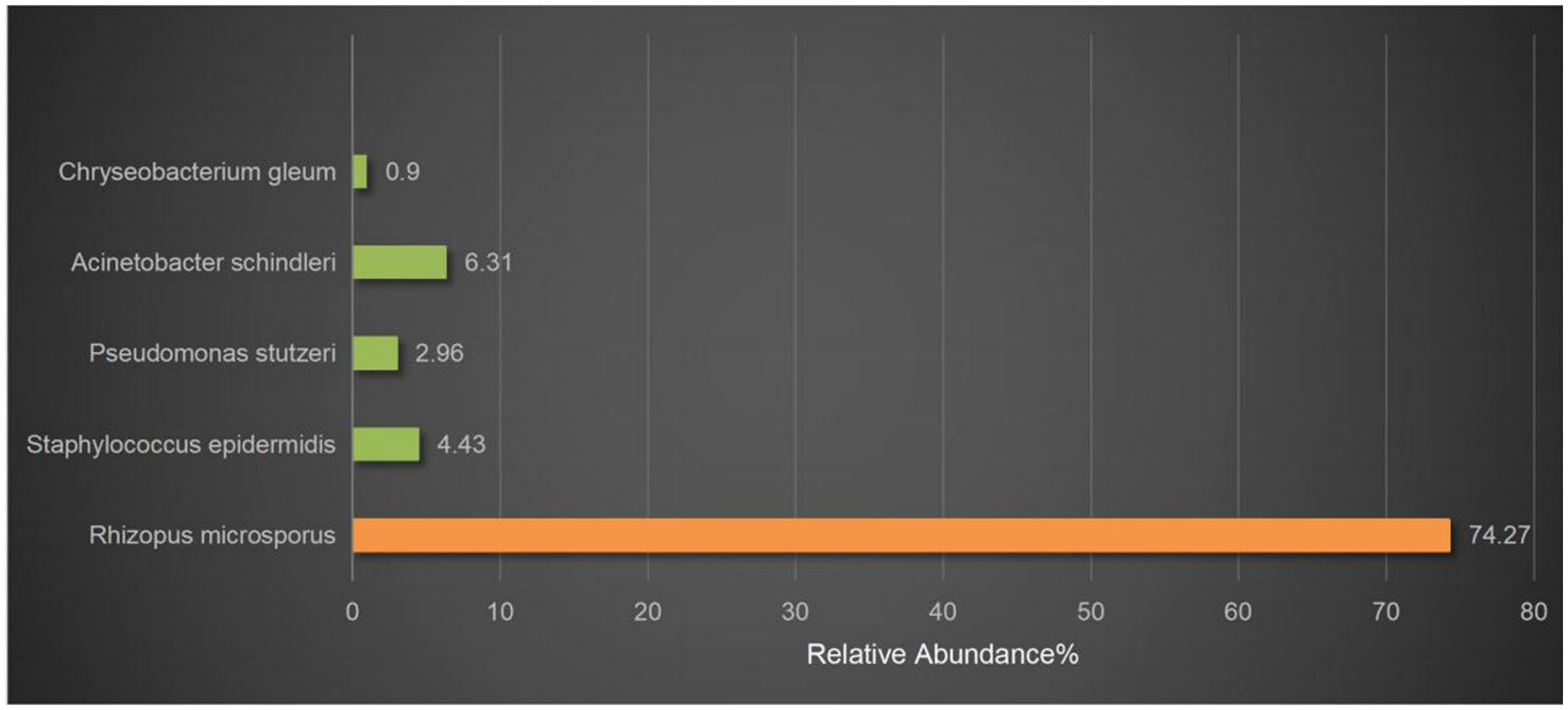
mNGS results. *Rhizopus* was detected by mNGS. The relative abundance of *Rhizopus* in all microbial was 74.27%. The others were considered probable normal flora.

**Figure 4:**
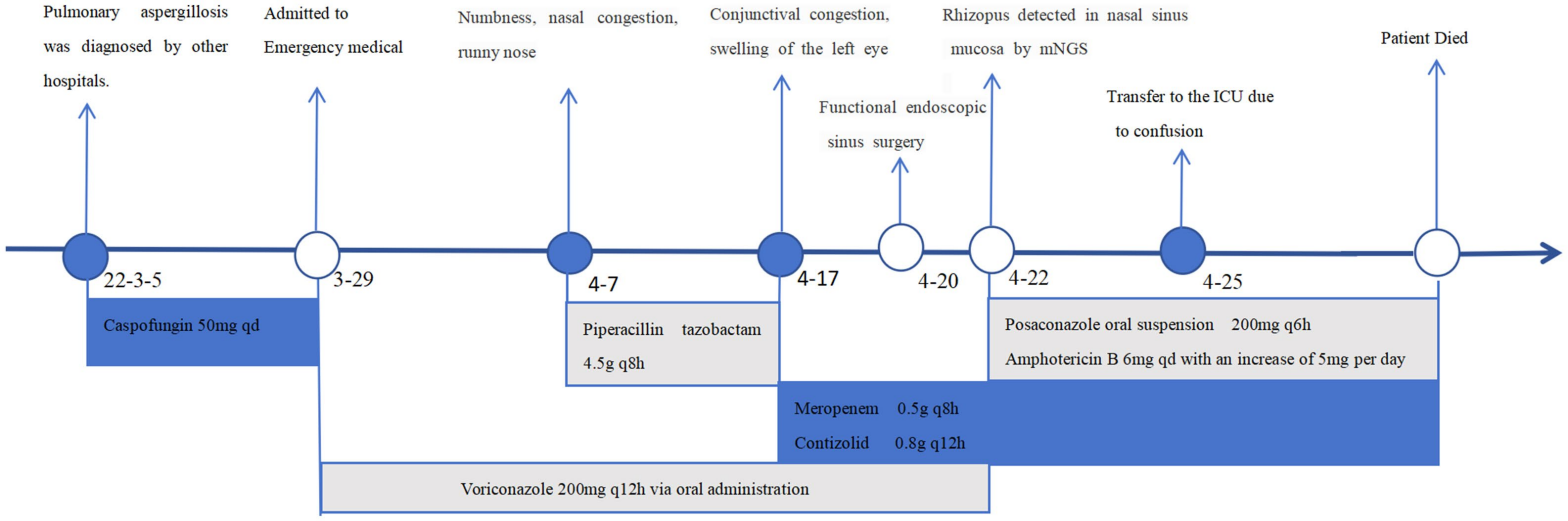
The treatment timeline of the patient.

On March 29, 2022, a 32-year-old male patient with systemic lupus erythematosus was admitted to the hospital due to recurrent cough for 1.5 months and abnormal kidney function for 1 month. The patient’s CT on February 22 suggested pulmonary infection, and multidisciplinary consultation in the outside hospital considered pulmonary aspergillosis (Figures 1A,B). After 23 days of treatment with caspofungin (50 mg qd), the patient’s lung condition improved (Figures 1C,D). Admission diagnosis: Systemic lupus erythematosus, lupus nephritis type IV + V, pulmonary aspergillosis. The patient received methylprednisolone and cyclosporine to control the primary disease, and voriconazole was taken orally to treat pulmonary aspergillosis after admission. On April 7th, the patient developed numbness in the left cheek, nasal congestion, runny nose, and fever. Endoscopic examination showed pus in the left middle meatus and blood scab on the surface of the middle turbinate (Figure 2A); CT showed left ethmoid and frontal sinusitis (Figure 2B). Two cultures of nasal secretion yielded no bacterial or fungal growth. The patient received additional piperacillin tazobactam (4.5 g q8h) anti-infection and nasal irrigation. During this period, the patient’s platelets decreased progressively, with a platelet count of 30×10^9/L on April 16th. Considering the low platelet count of the patient, invasive treatment was not recommended. The patient’s antibiotic was changed to meropenem (0.5 g q8h) combined with contezolid (0.8 g q12h), and gamma globulin (20 g qd) was administered for 3 days to enhance immunity. On April 17th, the patient’s left conjunctival swelling and soft tissue protrusion worsened compared to before. On April 19th, the patient experienced sudden vision loss in the left eye at night. The patient underwent emergency functional endoscopic sinus surgery to clear the lesions in the maxillary sinus, ethmoid sinus, frontal sinus, and orbit (Figure 2C). Intraoperative pathology reported: a large number of fungal hyphae and spores were seen in the nasal and sinus mucosa (Figure 2D). mNGS of the infected tissue in the sinus during the operation: *Rhizopus* (Figure 3). Following the surgery, the antibiotic regimen was modified to include meropenem (0.5 g q8h), contezolid (0.8 g q12h), amphotericin B 6 mg daily with a daily increase of 5 mg, and posaconazole suspension (200 mg q6h). The patient’s vision in the left eye showed some recovery 1 day after the surgery. Nevertheless, the patient suffered from severe agranulocytosis and extreme immunosuppression, resulting in an extremely weakened mental state and gradually declining consciousness. CT on April 25th showed brain parenchymal swelling, brainstem swelling and fuzzy, multiple low-density foci in the brain, and mild right shift of the midline, indicating intracranial infection with brain herniation formation. Eventually, the patient died due to systemic infection and multiple organ failure. The entire treatment process is summarized in Figure 4.

## Discussion

*Mucor*, an opportunistic pathogenic fungus second only to *Aspergillus* and *Candida*, is widely present in nature, such as in the air, soil, plants, and rotten substances. It is an airborne pathogen, so most mucormycosis cases initially occur in the sinuses and lungs (4). In the mid-20th century, diabetes was a major risk factor for mucormycosis. In recent years, more cases have been reported in immunocompromised groups, including those undergoing chemotherapy or cancer immunotherapy, and solid organ and hematopoietic stem cell transplantation (5). Cutaneous and soft tissue mucormycosis is the most common form in immunocompetent patients, mainly after skin damage from traumatic injuries, surgery or burns (6). *Mucor* has vascular invasiveness. After invading blood vessels, it can cause thrombosis and vascular occlusion. This leads to ischemia and hypoxia of local tissues, and then necrosis occurs.

RCM can spread through blood vessels to the sinuses, eyes, and brain. Early symptoms are nonspecific, such as nasal congestion, swelling and pain, or headache with fever. Untreated, it can spread to orbit and brain in a few days, causing edema, vision loss, and drowsiness. Severe cases lead to paralysis, coma, or death. RCM can be divided into four stages based on lesion scope: Stage I is only the nasal cavity; stage II involves the ipsilateral sinus and orbit; stage III is the brain with no or limited cognitive impairment; and stage IV is bilateral nose, orbit and brain with loss of consciousness or hemiplegia (7). Meningitis, large vessel septic thrombophlebitis, septicaemia and cerebrovascular accident are common causes of death.

The patient in our study was diagnosed with RCM. Upon admission, the patient was on antifungal treatment for pulmonary *Aspergillus* infection. Initially, sinusitis was thought to be caused by *Aspergillus* or bacterial infection. The patient’s nasal infection progressed rapidly. Nasal secretion culture and other routine examinations yielded unclear results. Due to a low platelet count, nasal histopathological examination was not promptly performed. When the patient had a sudden drop in visual acuity, we performed active surgical debridement after communicating with the patient’s family. Intraoperative biopsy found fungal hyphae and spores but could not identify the fungus type. To determine the pathogen causing the infection, mNGS was carried out on the sample. The results showed that the patient was infected with *Rhizopus*. Fungal culture and antifungal susceptibility testing can help guide clinical treatment, but they have a low detection rate and are time-consuming (8). Diagnosing mucormycosis on histomorphological basis is challenging. The *Aspergillus* genus and the *Mucor* genus have differences in morphology: the hyphae of the former are thinner and septated, with branches at approximately 45 degrees; the hyphae of the latter are wider and non-septated, with branches at approximately 90 degrees (9). However, a definitive diagnosis cannot always be made based on tissue fungal morphology due to similarities and sparse or atypical elements (10). The most common morphological misdiagnosis is misidentifying *Mucorales* as *Aspergillus* spp. (11).

The advantages of mNGS sequence-based detection of microbes associated with a disease state include high-throughput assessment, simultaneous detection of bacterial, fungal, and viral community members, and reliable sequence data quickly (12). However, its relatively high cost may limit its application in regions with limited medical resources. Also, mNGS cannot conduct drug sensitivity tests. For diagnosing fungi with mNGS, deep tissue samples are needed to avoid contamination or isolation of pathogenic saprophytes (13). Moreover, the operation and data analysis of mNGS require professional techniques and knowledge, and there are certain requirements for the technical level and personnel quality of medical institutions. Currently, it cannot replace traditional methods. It might be recommended for high-risk patients, who can benefit from early diagnosis and treatment. Despite these challenges, with technological progress and cost reduction, mNGS is expected to become a popular diagnostic tool.

The *Aspergillus* infection in the patient’s lungs improved after being treated with voriconazole. However, on the 9th day after hospitalization, the patient developed nasal symptoms, and dark purple crusts were found during nasal endoscopy, raising the possibility of nosocomial infection. Previous literature has also suggested that the increase in the number of infection cases of mucormycosis is due to the increased use of anti-Aspergillus drugs (14).

*Rhizopus* invading blood vessels can form blood clots, causing ischemia and necrosis of surrounding tissues. Antifungal drugs are difficult to reach the infected site, so surgical debridement is crucial. Nasal mucormycosis has typical endoscopic manifestations, such as dark purple crusts or pale mucosa (Figure 2A). Vaughan et al. found that the survival rate of surgical treatment within 12 days of onset of RCM is 61, 33% after 12 days, and only 21% for those without surgical treatment (15).

Voriconazole is currently recommended as the first-line treatment for invasive aspergillosis but not for invasive mucormycosis (10, 16). Posaconazole oral suspension and amphotericin B have been used to successfully treat mucormycosis with various organ involvement patterns as first-line treatment (10, 16). In addition, isavuconazole has been licensed in the USA for first-line treatment of mucormycosis (17). Amphotericin B carries the risk of hypokalemia and renal damage, so attention should be paid to checking potassium ions and renal function. The main adverse effect of posaconazole is hepatotoxicity, which may cause shortening of the QTc interval. In animal models, some antifungal combinations have shown the potential to improve cure and survival rates with no antagonism noted (18). In trauma patients, particularly those with blast injury, mixed infections involving multiple species may occur, warranting empirical combination therapy with liposomal amphotericin B and either posaconazole or voriconazole (19). In our case, the patient had concurrent *Aspergillus* pulmonary infection and was treated with a combination of posaconazole oral suspension and liposomal amphotericin B.

RCM has a rapid onset and progression, and clinicians have relatively low awareness of this disease. In our case, the patient had *Aspergillus* pulmonary infection complicated with RCM, and the nasal infection pathogen was not detected in time by conventional tests. Due to the patient’s low immunity, intracranial infection eventually led to death. This case highlights the advantages of mNGS in detecting rare and mixed infection pathogens, which may help in the diagnosis of infectious diseases.

## Data Availability

The original contributions presented in the study are included in the article/supplementary material, further inquiries can be directed to the corresponding author.
